# Design and analysis of capacitive pressure sensor with L shaped electrode for enhanced sensitivity

**DOI:** 10.1038/s41598-025-14964-3

**Published:** 2025-08-13

**Authors:** Bhagya R. Navada, Nanditha Nair, Santhosh Krishnan Venkata

**Affiliations:** Department of Instrumentation and Control Engineering, Manipal Institute of Technology, Manipal Academy of Higher Education, Manipal, India

**Keywords:** Capacitance, Fabrication, Pressure sensor, Sensitivity, Range, Engineering, Electrical and electronic engineering

## Abstract

Accurate pressure measurement is essential across a wide range of fields, including engineering, healthcare, and transportation. This paper presents the design and development of a novel capacitive pressure sensor featuring an extended measurement range and a large pressure application area compared to the parallel plate capacitor configuration. The main innovation lies in the shape and structure of the L shaped electrode which enhances the operational range in terms of the displacement, increased surface area. The sensor exhibits enhanced sensitivity and operational efficiency, making it well suited for diverse applications. The sensor is modeled and simulated in COMSOL MULTIPHYSICS environment to validate its functionality under varying pressure. The electric field distribution, change in capacitance and change in displacement are measurable from the simulation. The sensor is capable of bidirectional making it suitable for the measurement of differential pressure. The proposed sensor simulation results achieve an 11% improvement in both sensitivity and range over conventional parallel plate designs. Furthermore, its ability to operate as a bidirectional device highlights its adaptability and potential for integration into advanced sensing systems.

## Introduction

A capacitive pressure sensor is a pressure-sensing device that uses a diaphragm and a cavity to construct a flexible capacitor. This capacitor undergoes variations in capacitance due to the strain induced by the applied load or pressure. The pressure applied results in an alteration in the capacitor’s surface area or the distance between the plates, consequently leading to a change in the capacitance. This capacitance change is measured using a bridge circuit or a multivibrator circuit. Capacitive pressure sensors are widely utilized in various applications, including the measurement of gas or liquid pressure within the human body, as well as in airplanes, jet engines, and wearable devices. They are also employed to gauge applied pressure on components such as keyboards or switches in different settings.

The principle of capacitance is used in designing pressure sensors, and few studies are stated below. In^1^, works related to nanomaterials such as carbon used as dielectrics and metals used as electrodes, such that capacitance pressure sensors are formed, are discussed. Various capacitance pressure sensor designs and structures, such as electrode shapes and materials used for the fabrication of electrodes and dimensions, are reviewed in^2^. The design of a capacitance pressure sensor via MEMS technology with a multilayered electrode was reported in^3^, and the multilayer structure of the electrode was used to increase the linearity of the pressure sensor. Furthermore, in^4^, the hierarchical structure of the electrode led to improved sensitivity of the capacitance pressure sensor. In^5^, it is claimed that using a porous ion membrane in capacitance pressure sensor fabrication would lead to enhanced sensitivity. The design of a printed carbon nanotube-based capacitance pressure sensor for improved sensitivity was reported in^6^. Polymers are used as dielectrics with silver electrodes printed in^7^ to form capacitance pressure sensors to achieve higher resolution with decreased offset current for application in the biomedical field. Similarly, in^8^, printed sensor electrodes were used to design capacitance pressure sensors for analyzing pressure points to monitor chronic wounds. Capacitance pressure sensors are designed with electrodes knitted in the form of fabric to achieve a greater surface area and thereby increase the resolution of the sensor^9^. Electrodes, such as tooth-like structures in capacitance pressure sensors, are designed to improve the sensitivity and sensing range, as they increase the surface area^10^. A microplate array is used to design a pressure sensor as the capacitance changes when the electrodes tilt due to changes in pressure^11^. Similarly, a micro pyramid structure is used to design a capacitance pressure sensor for improved sensitivity for use in biomedical applications^12^. A pressure sensor used in robotic applications leading toward health management with a microarray electrode structure for improved performance was reported in^13^. Copper nanofiber networks are used as electrodes in capacitance pressure sensors to increase the range and sensitivity of measured pressure^14^. Table 1 presents the techniques used in^15–29^ with their objectives.

**Table 1:** Comparison of previous literature studies.

Paper	Technique	Objective
^15^	Micropore electrode type capacitive pressure sensor	Improve response time
^16^	Use of composite carbon paste as dielectric of capacitive pressor sensor	Improve linearity and sensitivity
^17^	Design of capacitive electrodes with pyramidal structures	Improve range
^18^	Porous dielectric in Capacitive pressor sensor	Sensitivity and range improvement
^19^	Organic array of dielectric in capacitive pressor sensor	Sensitivity and range improvement
^20^	Variation in dielectric due to presence of organic liquid (blood)	Sensitivity improvement and there by measure blood pressure
^21^	Cone shape dielectric capacitance pressure sensor	Increase sensitivity
^22^	Polydimethylsiloxane dielectric for measuring biological parameter	Increased sensitivity and dynamic range
^23^	Array of graphene dielectric	Improved power consumption
^24^	Flexible nano structure electrode	Wide working range and increasing sensitivity
^25^	Diaphragm type capacitive pressure design with different shapes like circle, square, etc	Improved sensitivity and range
^26^	Pyramidal structure hyper elastic dielectric structure	Increased sensitivity, low range
^27^	Flexible capacitive pressure sensor using polydimethylsiloxane	Increase bending angle and improve temperature effect
^28^	Porous polymer dielectric capacitance pressure sensor	Improved sensitivity, bending and range
^13^	Micro design layered capacitor pressure sensor	Improved range and sensitivity
^29^	Textile based meshed multilayer fabric as dielectric in capacitive pressure sensor	Improved response reduced run time and improved sensitivity

Many researchers have worked in the field of designing capacitive sensors to improve their sensitivity and range through diverse shapes of conductors. In this study, the objective is to design a capacitive sensor that has a wider range of operation and good sensitivity in the operating range. The innovative L-shaped structural design of the proposed sensor is its novel feature; it increases the effective electrode surface area and significantly boosts sensitivity. Unlike conventional geometries, the L-shaped arrangement adds mechanical flexibility and optimizes the electrode-to-electrode interaction, allowing the sensor to change dimensions in response to pressure. The proposed sensor aims to increase sensitivity by incorporating an L-shaped structure with an increased electrode surface area. A key benefit of this sensor is its heightened sensitivity and ability to alter its dimensions in response to applied pressure.

## Materials and methods

### Capacitive pressure sensor

**Fig. 1:**
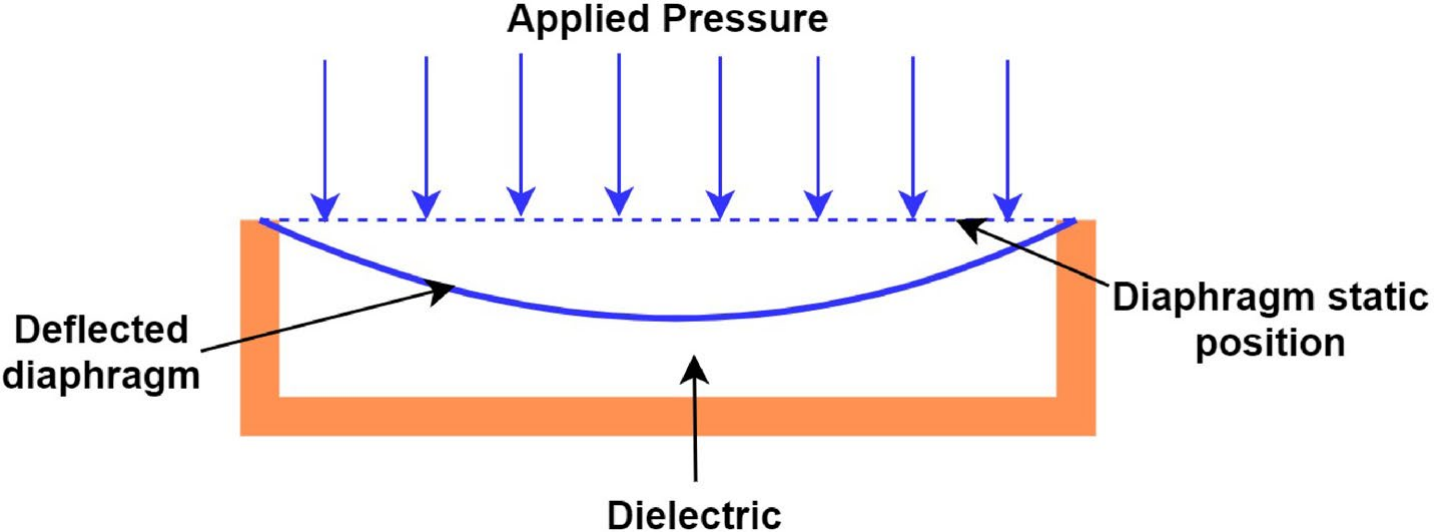
Basic capacitive pressure sensor.

A typical capacitive pressure sensor consists of a pair of parallel plates arranged to create a cavity, as shown in Fig. 1. When pressure is applied, the diaphragm deflects, and this deflection can be utilized to determine the magnitude of the applied pressure. A capacitive pressure sensor detects and reacts to externally applied pressure, with the specific pressure response determined by the design of the sensor. The calculation of capacitance in a parallel plate capacitor is performed using Eq. (1). When pressure is applied, the diaphragm deflects, altering the distance and overlapping area between the plates. Consequently, this modification leads to a variation in the measured capacitance. The relationship between the deflection of the diaphragm and the applied pressure is one of direct proportionality. Equation (1) expresses the relation of the capacitance between the plates as a function of the overlapping area and distance between the plates. The relationship shows that as the distance between the plates decreases, the capacitance increases, and when the area expands, the capacitance also increases.

1 $$C = \frac{{\varepsilon_{0} \varepsilon_{r} A}}{d}$$

where $\varepsilon_{0}$—permittivity of space in F/m, $\varepsilon_{r}$—relative permittivity of the dielectric material, $A$—overlapping area of plates in m^2^, $d$—distance between the plates in m,

The choice of diaphragm material, such as glass, ceramic, or plastic, can vary depending on the specific requirements. The selection of material thickness and stiffness is affected by the magnitude of the load exerted on the diaphragm. The shape of the diaphragm also influences the response of the sensor. To understand the response of different diaphragm shapes, identifying a model of various diaphragm shapes is imperative.

### Modeling

The plates are assumed to undergo deflection when a load is applied on them, and the deflection it undergoes is said to be in accordance with the “Kirchhoff–Love plate theory”.

Kirchhoff–Love Plate Theory:

The theory is based on small and large deflections in the diaphragm/plate. To apply the small deflection theory, the diaphragm deflection must be less than 1/5th of the diaphragm thickness.

Small deflection theory is based on Kirchhoff’s assumption, which is as follows:

- There is no deformation in the middle plane of the plate. This plate remains neutral during bending.
- Points of the plate lying initially normal to the middle lane of the plate remain normal to the middle surface of the plate after bending.
- The normal stresses in the direction transverse to the plate can be disregarded.

With these assumptions, all stress components can be expressed by the deflection ‘w’ of the plate, which is a function of the two coordinates in the plate.

### Flexural rigidity

When pressure is applied on a nonrigid structure, it undergoes external bending; at the same time, the material experiences an opposition force by the structure for the bending force. This force is termed the flexural rigidity of the material, and for a given plate, it is represented by Eq. (2).

2 $$D = \frac{{Et^{3} }}{{12\left( {1 - v^{2} } \right)}}$$

where *E*: Young’s modulus in Pa, *t*: the thickness of the diaphragm in m, *v*: Poisson’s ratio.

The deflection of the plate depends on the physical shape of the plate; thus, it is necessary to analyze the deflection in relation to the membrane geometry. Equation (3) represents the deflection of a square membrane; for a rectangular membrane, the deflection is provided in Eq. (4), and for a pentagonal membrane, it is represented in Eq. (5).

3 $$w_{square} = 0.00133\frac{{Pl^{4} }}{D}$$

4 $$w_{rect} = 0.0239\frac{{Pl^{4} m^{4} }}{{D\left[ {7\left( {l^{4} + m^{4} } \right) + 4l^{2} m^{2} } \right]}}$$

5 $$\left[ {\frac{{\partial^{4} }}{{\partial x^{4} }} + \frac{{2\partial^{4} }}{{\partial x^{2} \partial y^{2} }} + \frac{{\partial^{4} }}{{\partial y^{4} }}} \right]w\left( {x,y} \right) = \frac{P}{D}$$

where *P*—applied pressure, *l*- aide of the square/pentagon/shorter side of the rectangle, *m*—longer side of the rectangle, *D*—flexural rigidity in Pa.m^3^, $v$—Poisson’s ratio, $w\left( {x,y} \right)$—The deflection at point (x, y) of the plate.

Applying the boundary conditions and finding the maximum deflection due to the application of pressure at the center of the pentagon; thus, x = y = 0 results in Eq. (6):

6 $$w_{p,o} = 0.0041\frac{{Pl^{4} }}{D}$$

From Eq. (6), it is inferred that the maximum deflection is a function of the dimension of the plate, applied pressure, material properties and plate thickness.

### Comparison of deflection of different shape plates

The deflection ratios of the square, rectangular and pentagonal diaphragms are compared to observe the suitability of the diaphragm shapes for various applications. To compare the deflection among different shapes, it is necessary to maintain the same area of the plates. The dimensions of the square, rectangular and pentagonal plates are represented here so that the area of each of these plates remains the same.

*L*—Side of the square/shorter side of the rectangle = 40 mm,

*M*—Longer side of the rectangle = $2 \times l$ = 80 mm,

*q*—Side of the pentagon = $\frac{2 \times l}{{\sqrt {\sqrt {5(5 + 2\sqrt {5)} } } }}$ = 30.5 mm.

**Fig. 2:**
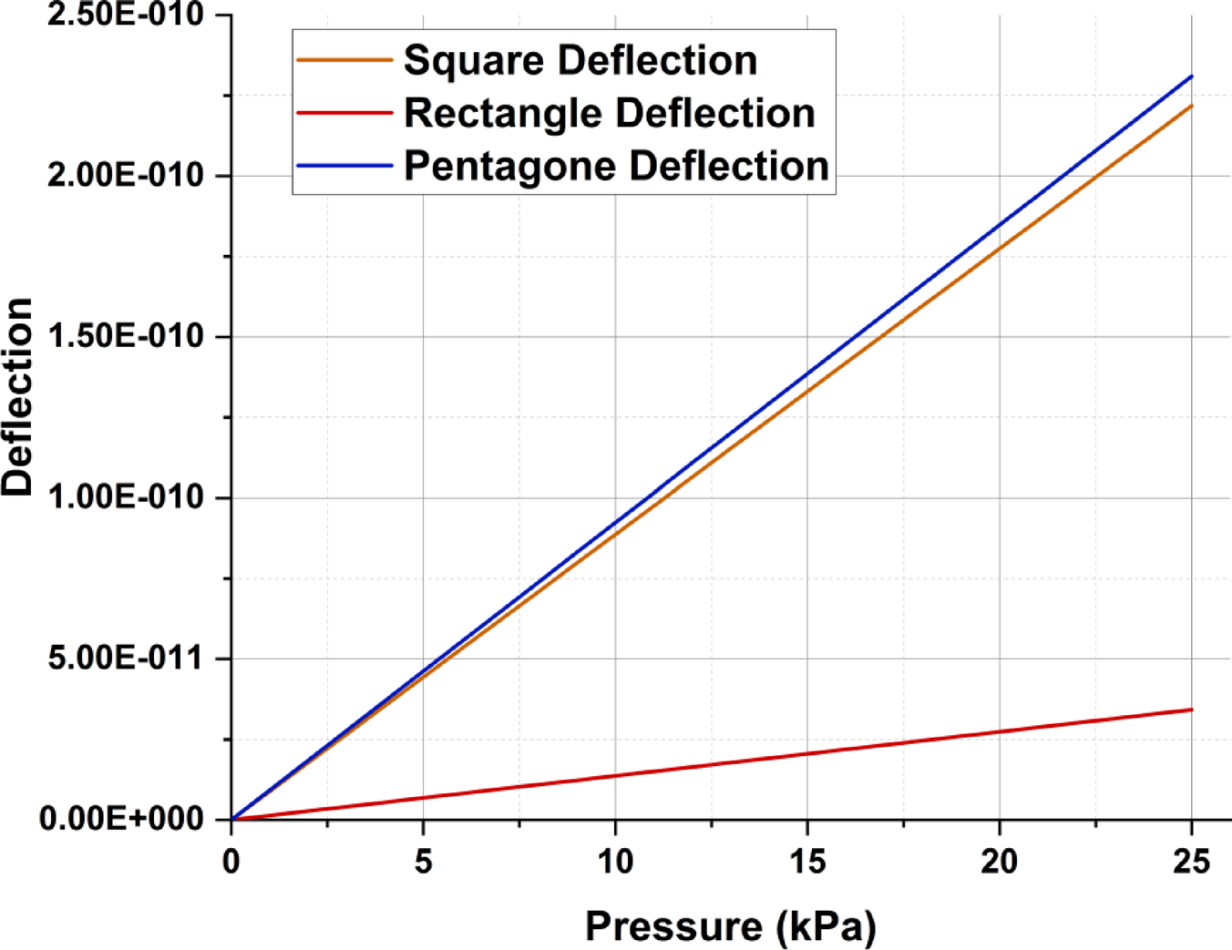
Comparison of different diaphragm shape deflections upon pressure application.

A comparative plot is depicted in Fig. 2, which plots the deflection of square, rectangular and pentagonal diaphragms when the pressure applied varies from 0 to 25 kPa. We observe that the deflections of the square and pentagonal diaphragms have relatively similar responses, whereas the rectangular deflection is relatively low. Hence, square plate diaphragms are best suited when a larger load‒deflection ratio is needed, and a rectangular diaphragm would be the best option for less capacitance change for increased loads. When the plates are subjected to pressure, they undergo deflection ‘w’, which reduces the plate separation gap ‘d’ by the amount of deflection. This change in the separation gap in response changes the capacitance, thus assisting in detecting the amount of applied pressure. The change in capacitance can be represented as given in Eq. (7).

7 $$C = \iint\limits_{A} {\frac{\varepsilon A}{{\left( {d - w} \right)}}}$$

8 $$C = \iint\limits_{A} {\frac{\varepsilon dxdy}{{d - w\left( {x,y} \right)}}}$$

9 $$C = C_{0} \left( {1 + \frac{{12.5Pl^{4} }}{2015Dd}} \right)$$

where $C_{0}$ is the initial capacitance when the pressure applied is zero.

The capacitance equations for squares, rectangles and pentagons have negligible differences in terms of the maximum deflection because all these geometric shapes have no difference for lower powers and disregard higher powers; the capacitance equation is approximated as given in Eq. (8). It is doubly integrated through both axes, representing the equation of a rectangle. By substituting the equation of deflection after double integration, the change in capacitance can be given by Eq. (9),

*Mechanical sensitivity* The mechanical sensitivity of a capacitive pressure sensor can be determined by differentiating deflection at the center, i.e., maximum deflection for the applied load or pressure. Below are the mechanical sensitivity expressions for different diaphragm shapes.

The mechanical sensitivity for a square or rectangular diaphragm can be given by Eq. (10):

10 $$S_{e,mech} = \frac{{\partial w_{e,0} }}{\partial P}$$

After the values are substituted and differentiated, the mechanical sensitivity for a square diaphragm is represented in Eq. (11), that for a rectangular diaphragm is represented in Eq. (12), and that for a pentagonal diaphragm is represented in Eq. (13).

11 $$S_{e,mech} = \frac{{0.00133l^{4} }}{D}$$

12 $$S_{e,mech} = 0.0239\frac{{l^{4} m^{4} }}{{D\left[ {7\left( {l^{4} + m^{4} } \right) + 4l^{2} m^{2} } \right]}}$$

13 $$S_{e,mech} = 0.0041\frac{{l^{4} }}{D}$$

*Capacitive sensitivity of diaphragms* The capacitive sensitivity of a CPS can be obtained by differentiating the capacitance change for the range of applied pressures, as represented in Eq. (14).

14 $$S_{e,cap} = \frac{{\partial C_{w,e} }}{\partial P}$$

Hence, for different diaphragm shapes, via the equation above, the capacitive sensitivity of all diaphragms can be rewritten as represented in Eq. (15).

15 $$S_{e,mech} = \frac{{C_{{{\text{max}}}} - C_{min} }}{{P_{{{\text{max}}}} - P_{min} }}$$

By designing electrodes for a capacitive pressure sensor, the proposed design addresses and resolves various issues present in previous designs. This is achieved by increasing sensitivity and incorporating a suitable casing for the dielectric. The utilization of a larger measurement area not only increases sensitivity and range but also ensures a consistent response over the entire operating range.

### Comparison of flexular rigidity due to changes in diaphragm material

The sensor response changes as the diaphragm material are altered. Several materials can be utilized as diaphragm materials based on these requirements. The most used diaphragm materials, such as silicon, aluminum, plastic, and ceramic, are compared based on the change in flexural rigidity. Poisson’s ratio, Young’s modulus and flexural rigidity of each material are presented in Table 2.

**Table 2:** Flexural rigidity of materials.

Material	Young’s modulus	Poisson’s ratio	Flexural rigidity (Pa m^3^)
PTFE	575 MPa	0.46	1.64
Aluminium foil	70 GPa	0.35	179.4
Silicon (111)	168.9 GPa	0.066	381.68
Ceramic aluminium oxide (Alumina al2o3, 96%)	303 GPa	0.21	713.2

Table 2 shows that Polytetrafluoroethylene (PTFE) has the least flexural rigidity, thus offering the least stiffness; hence, more deflection is allowed, followed by aluminum foil, silicon and ceramic aluminum oxide diaphragms. Ceramic aluminum oxide, which has maximum flexural rigidity, offers greater stiffness. When stiffness is the priority, ceramic diaphragms provide the best results; likewise, if the loads applied on the diaphragm are low, diaphragm materials that have low stiffness are needed; hence, PTFE or aluminum foil is preferred.

### Mechanical sensitivity

To compare the mechanical sensitivity of diaphragms with different shapes for silicon materials, a constant area for all shapes is imperative. Here, the sides of the square, rectangular and pentagon diaphragms are comparatively calculated to obtain the same area of the plates. The mechanical sensitivities of the diaphragms with different shapes of silicon material are calculated and presented in Table 3.

**Table 3:** Comparison of the mechanical sensitivity for different diaphragm shapes with silicon material.

Diaphragm shape	Mechanical sensitivity (nm/KPa)
Square	8.92
Rectangle	18.99
Pentagon	9.29

Table 3 shows that the mechanical sensitivity of the square and pentagon diaphragms is in the same range, whereas the rectangular diaphragm shows greater mechanical sensitivity. Thus, rectangular diaphragms are suitable in applications with the requirement of higher sensitivity.

From the observations of the changes in deflection and mechanical sensitivity with respect to changes in diaphragm shape, the rectangular diaphragm exhibited superior performance. Thus, in this work, a novel pressure sensor design is proposed for a larger range of operation and high sensitivity by linking two rectangular plates and forming a complex structure, as detailed in the next section.

### L-shaped capacitive sensor design

A capacitive pressure sensor is commonly employed in various processing industries for the detection of differential pressure. The proposed sensor aims to increase sensitivity by incorporating an L-shaped structure with increased electrode surface area. A key benefit of this sensor is its heightened sensitivity and ability to alter its dimensions in response to applied pressure. The proposed sensor involves the design of electrodes of the capacitance pressure sensor, in which the sensitivity is dependent on the surface area of the electrode as the surface area increases. A uniform response of the sensor throughout the operating range can be achieved with the proposed design.

**Fig. 3:**
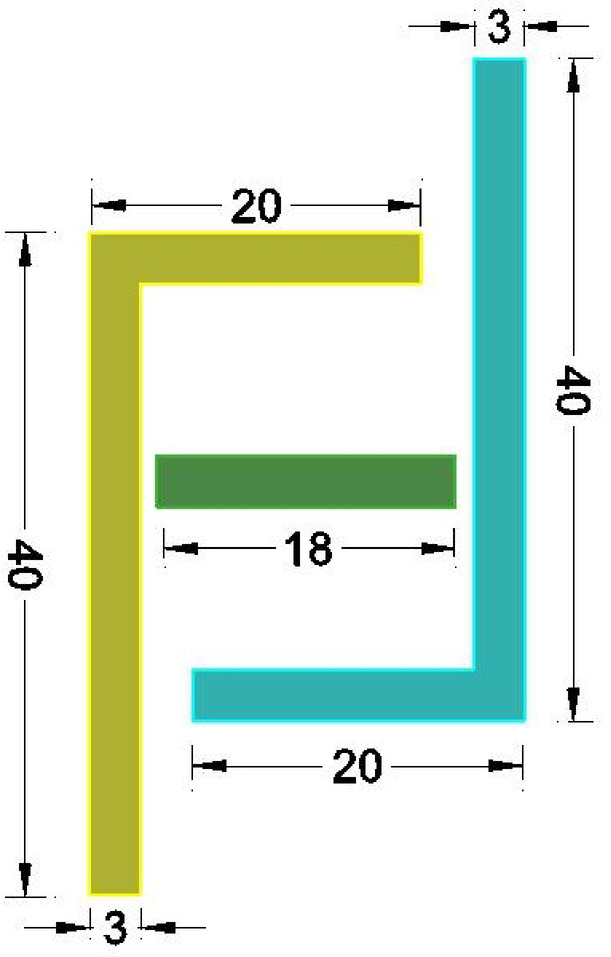
Top view of the L-shaped pressure sensor.

**Fig. 4:**
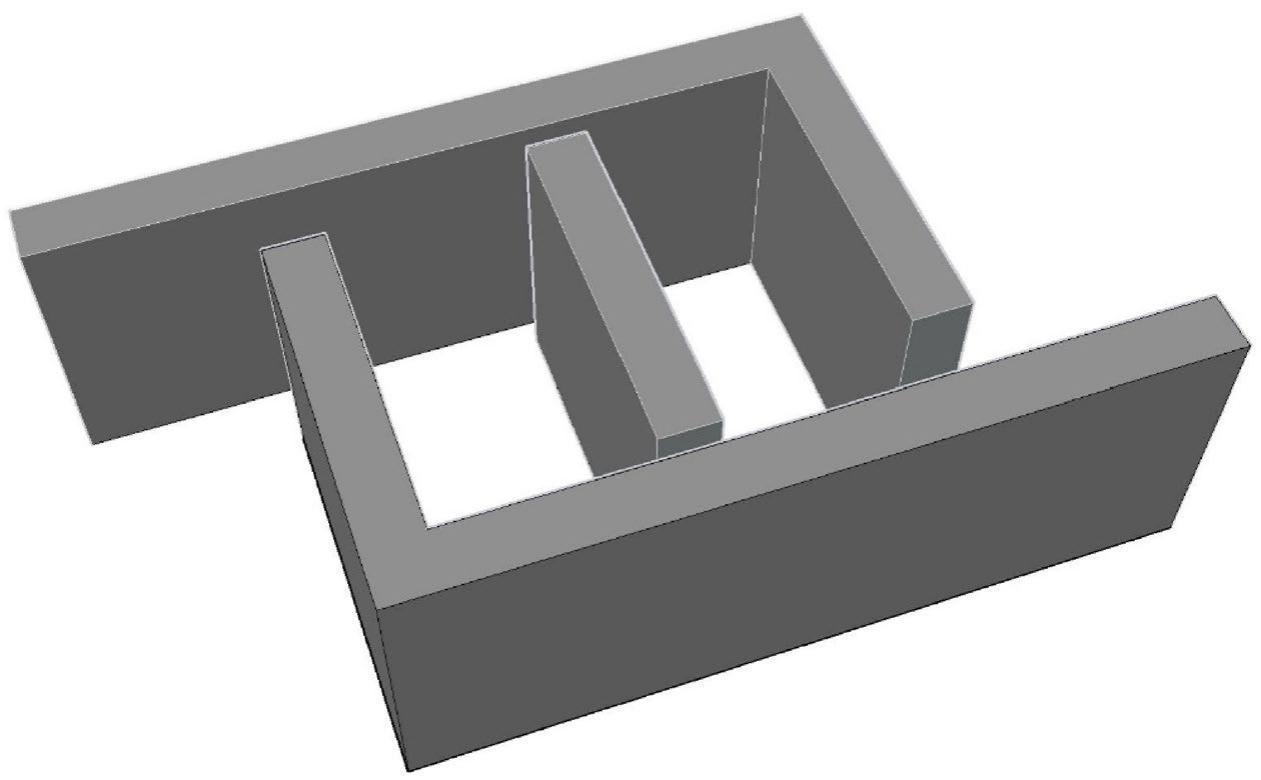
Isometric view of the 3D model of the L-shaped pressure sensor.

The sensor consists of two L-shaped diaphragms placed at opposite angles from each other and are placed at the same distance from the fixed reference plate, as depicted in Fig. 3, which shows the top view of the model designed in the AutoCAD tool. The dimensions of the sides of the L-shaped plates are tabulated in Table 4 and depicted in Fig. 3. The gaps between the reference plate and the shorter side of the L-shaped plate are approximately 10 mm on both sides. The depth of each side is 15 mm, which can be visualized in the isometric view of the sensor represented in Fig. 4. The metallic electrode is an L-shaped diaphragm that is made of aluminum material at the exterior of the sensor. To provide a reference for the movement of the electrodes, a rectangular cuboidal middle part is considered. As the diaphragm is L shaped, there is a scope for increased sensitivity to the applied pressure. The pressure is applied in the form of air; thus, a uniform pressure is applied on the shorter side of the L-shaped plates. When the moving area is large, the sensitivity to the applied pressure increases.

**Table 4:** Dimensions of the L-shaped capacitive pressure sensor.

Parameter	Dimension (mm)
Length of the long side of L-plate	40
Length of the short side of L-plate	20
The thickness of all the plates	3
Length of the reference plate	18
Depth of all the electrodes	15

**Fig. 5:**
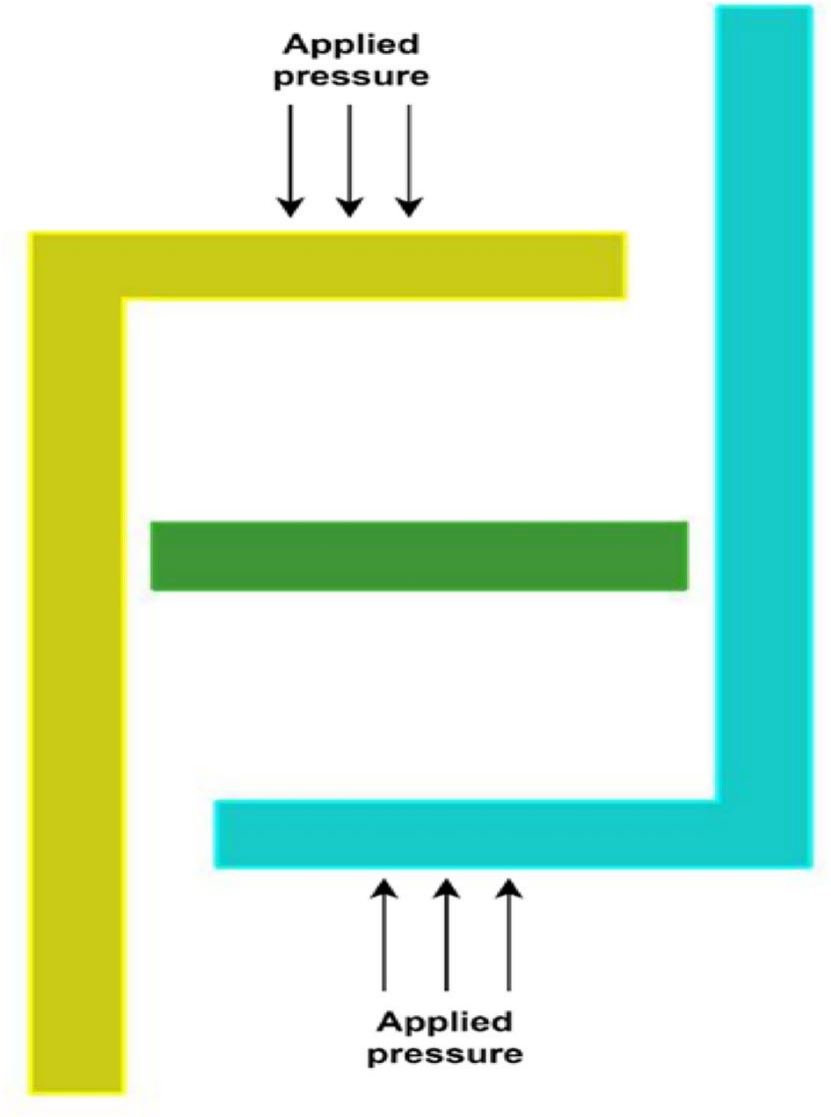
Top view of the L-shaped pressure sensor with an illustration of the direction of the applied pressure.

When the sensor is subjected to differential pressure, as denoted in Fig. 5, the plates begin moving toward or away from the reference plate, either reducing or increasing the gap between the reference plate and the L-shaped plate, respectively. As the capacitance of the proposed sensor is measured between the reference plate and the two L-shaped plates, the gap between the rectangular cuboidal reference part and the electrode influences the range of operation. The dielectric medium considered is air, with a relative permittivity of 1. The dimensions of the sensor also change as the plates start moving in relation to the applied pressure.

**Fig. 6:**
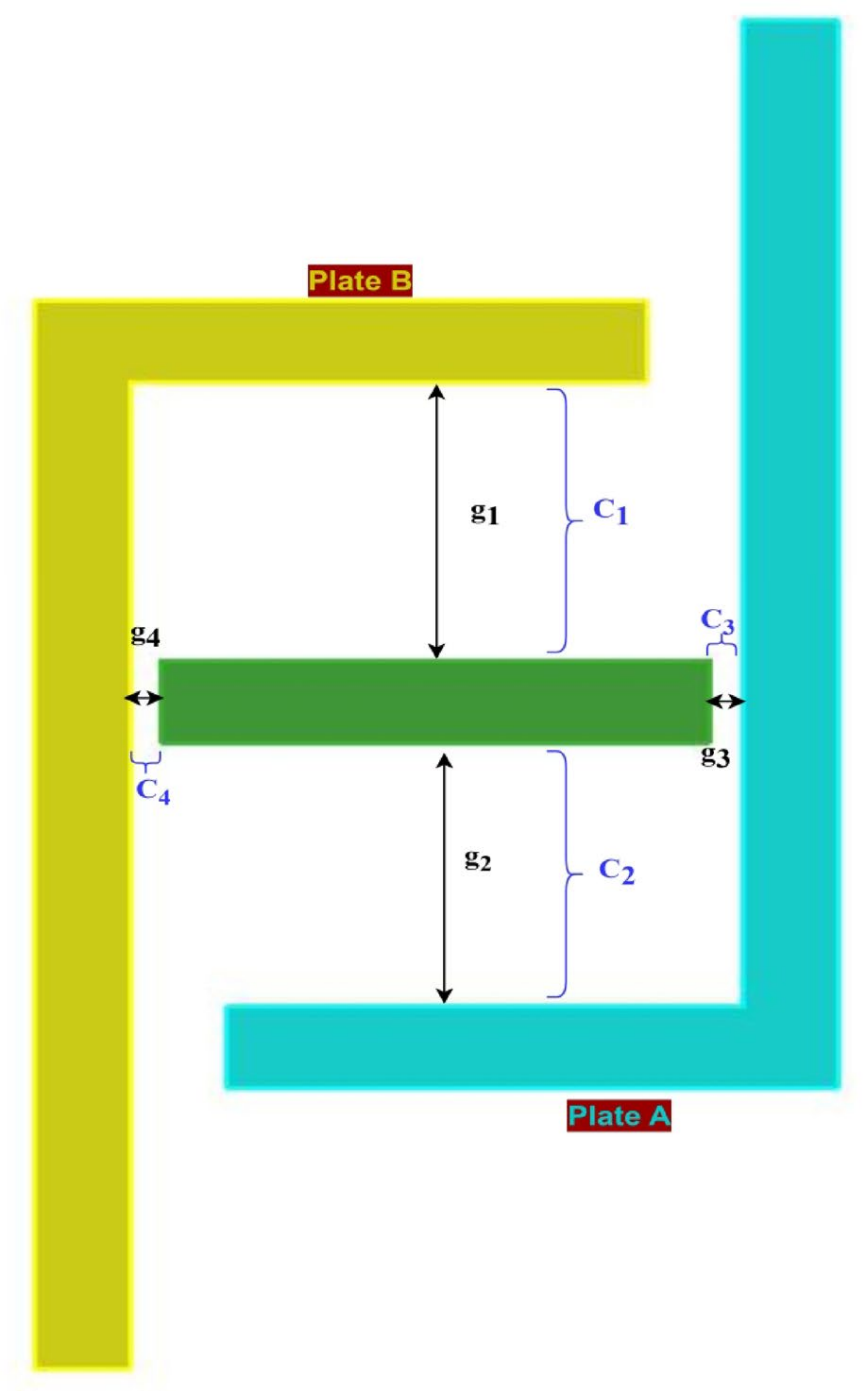
Representation of the gaps between different plates and the capacities contributing to the total capacitance between the two electrodes.

The total capacitance of the proposed L-shaped sensor is calculated by considering all the capacitances involved in the design. Four parallel plate combinations can be observed in the designed sensor. The representation of the gaps between different plates and the capacitances contributes to the total capacitance between the two electrodes, as represented in Fig. 6.

The distance and capacitance between the reference plate and plate A are ‘g_1_’ and ‘C_1_’, respectively. The distance and capacitance between the reference plate and plate ‘B’ are ‘g_2_’ and ‘C_2_’, respectively. The distances between the longer side of plate A and plate B and the reference plate are ‘g_3_’ and ‘g_4_’, respectively, where g_3_ = g_4_. The corresponding capacitances are considered constant and are represented as ‘C_3_’ and ‘C_4_’. The distances between the reference plate and the shorter sides of both plates are identical when no pressure is applied, i.e., g_1_ = g_2_ at pressure (P = 0). The fringe capacitances are assumed to be zero for theoretical calculations. Differential pressure is applied to both L-shaped plates, thus creating a change in capacitance based on the differential pressure applied. When differential pressure is applied, one plate tends to move away from the reference plate, whereas the other plate moves toward the plate. Plate A and plate B are connected to the same potential; thus, all the capacitances are parallel; thus, the total capacitance output from the sensor can be calculated as represented in Eq. (16).

16 $$C_{total} = C_{1} - C_{2} + C_{3} - C_{4}$$

where:$C_{3} = C_{4}$, C_total_ is the total capacitance between the reference and electrodes.

17 $$C_{total} = C_{1} - C_{2}$$

The total capacitance varies depending on the pressure applied on the plates.

The simulated values of total capacitance with the help of COMSOL Multiphysics software (version 5.5: https://www.comsol.com/release/5.5) are discussed in the Results section.

### Comparison with other capacitive pressure sensors

The sensitivity of the L-shaped sensor has been compared with the sensitivity attained by other similar sensors as shown in Table 5.

**Table 5:** Comparison of the sensitivity of the L-shaped capacitive sensor with other pressure sensors.

Paper	Sensor	Sensitivity
^30^	Capacitive pressure sensors with circular and square geometries	8.01 × 10^−5^ pF/Pa
^31^	Robust differential capacitive sensor with a grounded shield window	0.2 pF/mm
^32^	Cylindrical shell capacitive pressure sensor	2.312 × 10^−21^ pF∕Pa
Proposed sensor	L-shaped capacitive pressure sensor	1.68885 × 10^−3^ pF/Pa or 1.68885 pF/mm

It has been observed that the sensitivity attained by the L-shaped sensor is much better than the sensors that have been compared with it in terms of performance.

## Results and discussion

This section provides detailed discussion of the simulation results of the designed sensor using COMSOL Multiphysics software. COMSOL Multiphysics is a potent tool used in physics based model design and also analysis of the sensor behavior based on several parameter variations. This tool also provides the variation of important parameter like change in displacement, and change in capacitance for specific stimuli such as pressure, temperature in simulation.

### Capacitance simulation using COMSOL multiphysics

The proposed sensor behavior for the change in pressure in terms of the change in displacement is simulated in COMSOL Multiphysics software. The design discussed in the previous section was developed in COMSOL Multiphysics to analyze the sensor characteristics for the change in displacement of the two L-shaped plates. The developed sensor is placed inside a chamber with air; thus, the dielectric considered is air. The materials of the L-shaped plates and the reference plates are aluminum alloy, and the material of the casing is air. The designed sensor consists of two L-shaped plates with a reference plate forming four capacitances, as discussed in Eq. 17. The type of mesh applied is a physics controlled mesh with fine element size. The physics controlled mesh is used in order to ensure that the meshing procedure complies with the particular physics interface used in simulation. Based on the governing equations and the boundary conditions, it adjusts the mesh density thus more accurate results are obtained. Initially, the plates are placed at 5 mm from the reference electrode. The reference electrode is the fixed plate, whereas the L-shaped plates are movable based on the applied pressure. The pressure applied on the plates causes displacement, thus changing the distance between the plates and changing the final capacitance. In the simulation, the variable ‘d’ is varied from 0 to 4.5 mm and is the variable used for causing the change in displacement between the plates.

**Fig. 7:**
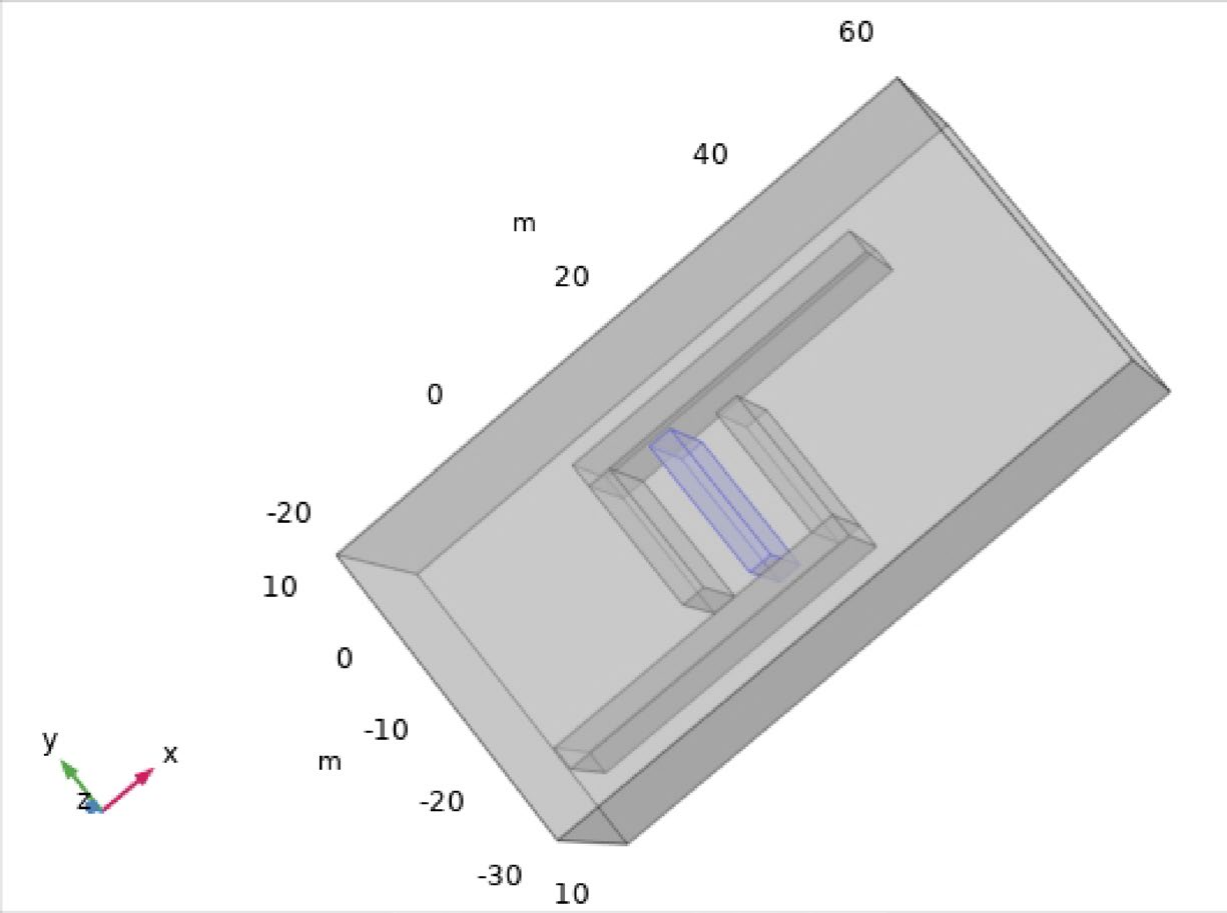
Geometry of the proposed L-shaped sensor in COMSOL Multiphysics.

**Fig. 8:**
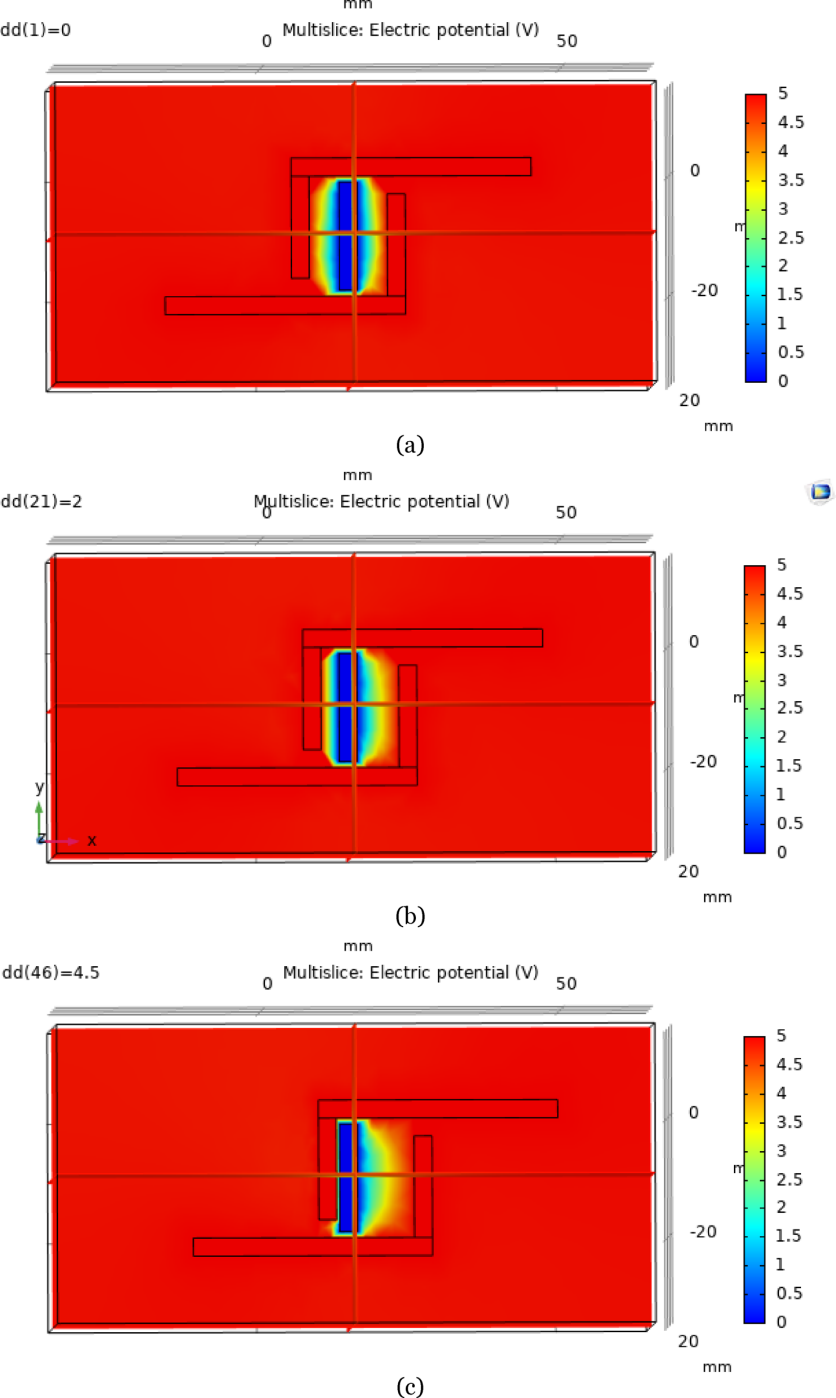
Potential distributions of the sensor at (**a**) d = 0, (**b**) d = 2, and (**c**) d = 4.5.

The sensor in COMSOL Multiphysics is shown in Fig. 7, and after the terminals are applied to the plates, the charge distribution is shown in Fig. 8a at d = 0. The distance between the plates is 5 mm at the beginning, and differential pressure is applied on the plates. The distance between the plates is varied such that as d increases, the distance between the reference plate and one of the plates changes by 5 d, and the distance between the reference plate and the other plate changes by 5 + d. The variable ‘d’ varies from 0 to 4.5 in steps of 0.1, and the capacitance values are calculated from the global evaluation. The charge distributions for d = 2 and d = 4.5 are also represented in Fig. 8b, c, respectively.

### Sensor capacitance comparison

To compare the capacitances of the parallel plate capacitor and the L-shaped capacitor, four cases are considered for calculating the total capacitance from the sensor.

#### Case 1: no pressure condition

Under no pressure conditions, the total capacitance is given by Eq. (18):

18 $$C_{totl} = 0$$

Here, when g1 = g2, the capacitance is $C_{1}$ = $C_{2}$, and when $C_{3}$ = $C_{4}$, the total capacitance becomes zero. The capacitance values are calculated via Eq. (19).

19 $$C_{total} = \left( {\frac{{\varepsilon_{0} \varepsilon_{r} A_{1} }}{{g_{1} }}} \right) - \left( {\frac{{\varepsilon_{0} \varepsilon_{r} A_{2} }}{{g_{2} }}} \right)$$

where $\varepsilon_{0}$ = 8.854X10-12 F/m, $\varepsilon_{r}$ = 1, A_1_ = A_2_ = 15 mmX16 mm = 240X10-6 m2, A_3_ = A_4_ = 3 mm X 15 mm = 45X10-6 m2, g_1_ = g_2_ = 5X10-3 m, and g_3_ = g_4_ = 1 X10-3 m, $C_{total}$ = 0 F.

#### Case 2: differential pressure applied on both plates

**Fig. 9:**
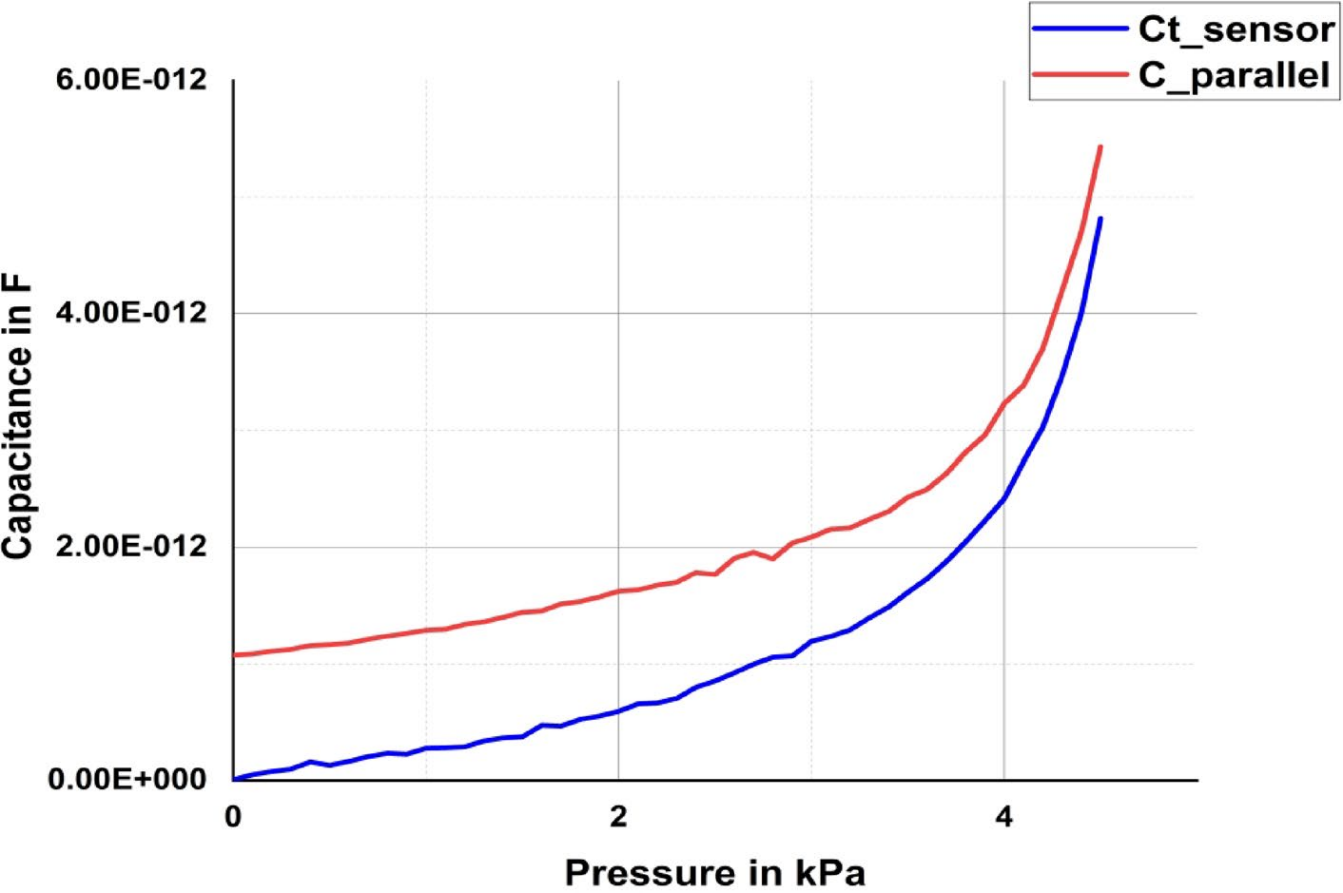
Comparison of the simulated L-shaped sensor with the parallel plate sensor.

In this case, both plates A and B move on the basis of the applied pressure. When pressure is applied to the plates, the plates start moving toward or away from the reference plate, reducing or increasing the distances g_1_ and g_2_. In this case, both plates A and B move on the basis of the applied pressure. Both L-shaped plates are connected to a positive voltage, and the reference plate is considered the reference ground. When differential pressure is applied to these plates, based on the pressure change, the capacitances related to plate A or plate B either increase or decrease, thus increasing the total capacitance. Here, as pressure is applied on both sides, the differential pressure creates a larger capacitance C_total_. Hence, the capacitance is directly proportional to the applied pressure. The total capacitance can be calculated via Eq. 17, where C_1_ is calculated for varying g_1_ and C_2_ is calculated for varying g_2_. Here, the distance between the plates is varied such that one plate moves toward the reference plate and the other moves away from the reference plate. In this case, both C_1_ and C_2_ vary with respect to the applied pressure. As the distance g_1_ decreases and g_2_ increases, the total capacitance gradually increases, as represented in Fig. 9.

We consider a parallel plate capacitor of area A_1_ and apply the same amount of pressure on both plates (moving plate and reference plate). The capacitance obtained by a parallel plate capacitor by applying pressure on the reference and moving plates is represented as C_parallel, and the capacitance of the proposed sensor is represented as Ct_sensor. The change in capacitance for applied pressure is plotted in Fig. 9 for both the parallel plate capacitor and the proposed L-shaped capacitor. The figure shows that the sensitivity of the proposed L-shaped capacitor is greater than that of the parallel plate capacitor for the same plate area and distance. The sensitivity of the parallel plate capacitor is 1.52282 × 10^−12^ F/kPa, whereas the sensitivity of the proposed sensor is 1.68885 × 10–12 F/kPa. Thus, the sensitivity of the proposed sensor is approximately 11% greater than that of the conventional parallel plate capacitor.

**Fig. 10:**
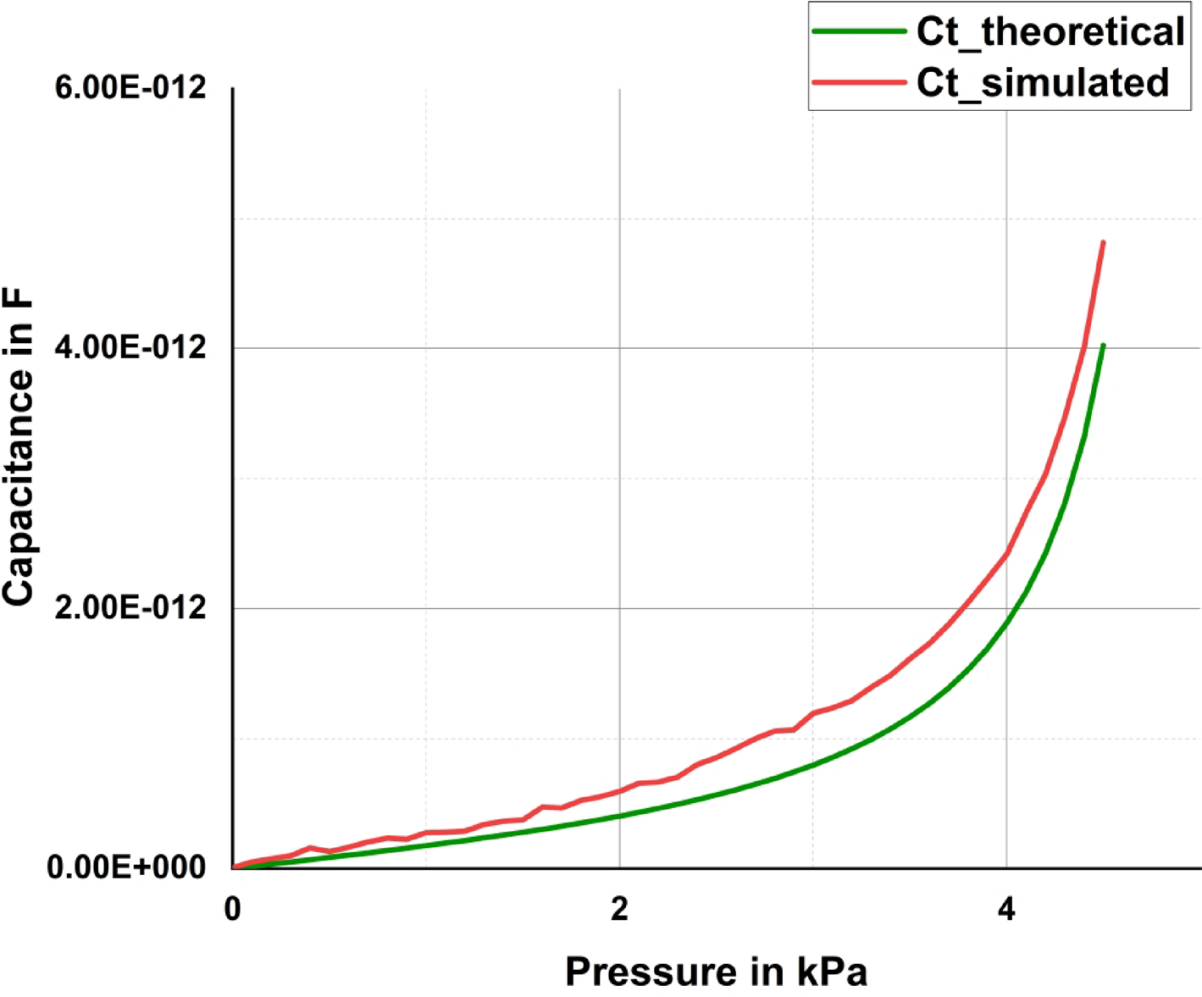
Comparison of the simulated capacitance with the theoretical capacitance of the proposed sensor.

The sensor is simulated in COMSOL Multiphysics software for a change in pressure of 0 to 4. 5 k Pa in terms of the variation in the distance between the plates from 0 to 4.5 mm. The capacitance obtained from the simulation and the theoretical calculated capacitance are plotted in Fig. 10. The figure clearly shows that the theoretical capacitance and the simulated capacitance follow the same trend.

### Analysis of sensor behaviour

The sensor behavior is analyzed for change in environmental conditions and also for change in dielectric medium. Firstly, the sensor behavior for change in dielectric medium is carried out in COMSOL Multiphysics through performing simulations using transformer oil and mica as the tw0 alternatives.

**Fig. 11:**
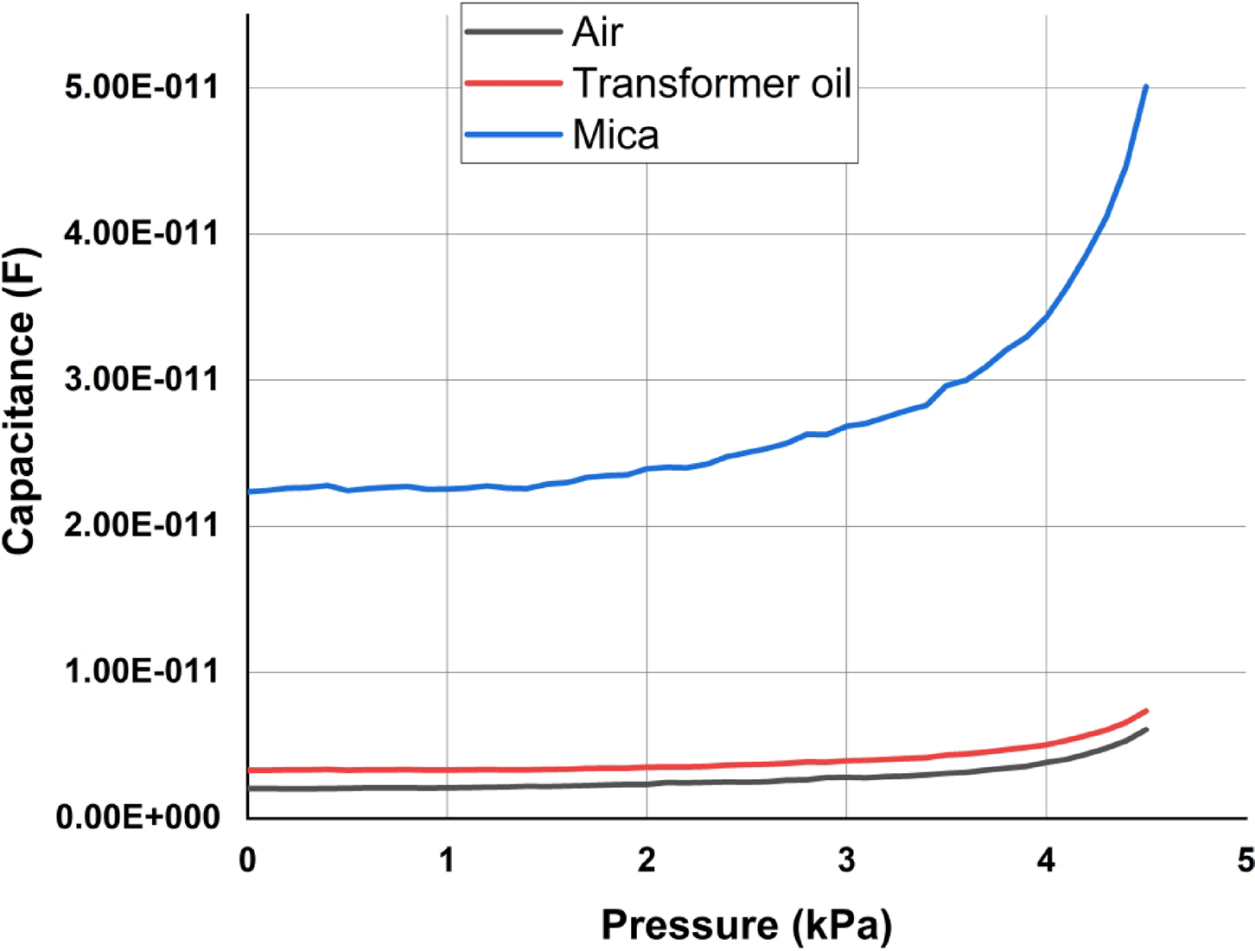
Response of the sensor for change in dielectric material.

To analyze the sensor behavior with different dielectric materials, the simulations were performed using transformer oil and mica as the tw0 alternatives. The transformer oil has a relative permittivity of 2.2 whereas mica has 6.8. As it is evident from the capacitor equation, an increase in relative permittivity results in increase in capacitance. From Fig. 11 it is observed that there is a shift in the capacitance when dielectric is changes whereas the sensor characteristics remain consistent.

To observe the effect of temperature on sensor behaviour, heat transfer properties are considered. Slight variations in sensor characteristics may occur with changes in temperature, as both the electrode material and the dielectric exhibit minor temperature-dependent property changes. The temperature is varied from 20 to 120 °C as the sensor is designed to use in environment conditions practically where the maximum temperature would not go beyond 100 °C. The sensor capacitance observed is 1.649 × 10^–12^ F at 20 °C and 1.649 × 10^–12^ F at 120 °C at a distance of 5 mm with reference plate. The difference in capacitance is at 11^th^ decimal place thus showing negligible effect of temperature on dielectric and the capacitor plate.

In this study, a uniform pressure was initially applied. However, a non-uniform pressure distribution was also simulated, which yielded similar sensor characteristics. It is important to note that in practical applications, variations in pressure distribution and the characteristics of the measurement device may lead to an increased response time.

**Fig. 12:**
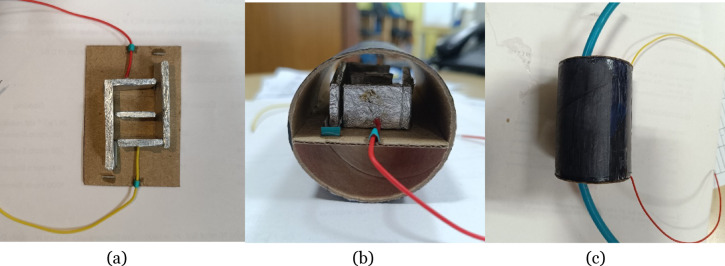
Proposed sensor (**a**) sensor design, (**b**) sensor (**c**) sensor with pressure inputs.

The proposed sensor is fabricated, and the corresponding images are shown in Fig. 12. The designed sensor is placed inside a case to apply pressure so that the sensor plate moves according to the applied pressure.

## Conclusions

This article presents the design of a hybrid L-shaped capacitive pressure sensor for the measurement of large-area pressure. When the change in capacitance of the proposed sensor is compared with that of conventional parallel plate capacitors, the advanced capacitive pressure sensor presented in this study has remarkably improved sensitivity, accuracy, and operational efficiency. The parallel plate capacitor has a sensitivity of 1.52282 × 10⁻^12^ F/kPa, whereas the proposed sensor has a sensitivity of 1.68885 × 10⁻^12^ F/kPa. This finding indicates that the proposed sensor is approximately 11% more sensitive than the conventional parallel plate capacitor. The sensor is also designed in COMSOL Multiphysics, and the capacitance values are obtained compared with the theoretical data where the sensor characteristics are similar.

Its high sensitivity and large range make it a suitable tool for diverse applications in engineering, healthcare, and transportation. These advancements not only expand the applicability of sensors to more challenging environments but also contribute to the progression of capacitive sensing technologies. The fringe capacitance is not considered to be a dominant parameter in this study; thus, all the calculations were performed under the assumption of negligible fringe capacitance. If the applied pressure exceeds the allowable range resulting in mechanical failure. Also the sensor response as can be observed from the graph is nonlinear in some range, thus can gives best results in allowable range. Future work will focus on the development of signal conditioning circuits to facilitate efficient processing of the measured values, paving the way for further integration and functionality in next-generation systems.

## Data Availability

The datasets generated during and/or analysed during the current study are available in the Sensor data repository, 10.17605/OSF.IO/PC87D.
